# Synthesis and Anti-Hepatitis B Activities of 3′-Fluoro-2′-Substituted Apionucleosides

**DOI:** 10.3390/molecules27082413

**Published:** 2022-04-08

**Authors:** Martin Holan, Kathryn Tucker, Natalia Dyatkina, Hong Liu, April Kinkade, Guangyi Wang, Zhinan Jin, Marija Prhavc

**Affiliations:** Janssen Research and Development, LLC, South San Francisco, CA 94080, USA; martin.holan@boehringer-ingelheim.com (M.H.); ktucker1@its.jnj.com (K.T.); ndyatkin@its.jnj.com (N.D.); rainbowforever001@gmail.com (H.L.); april.kinkade@gmail.com (A.K.); gyiwang1@gmail.com (G.W.); zjin14@its.jnj.com (Z.J.)

**Keywords:** HBV-antivirals, apionucleosides, DNA-polymerase inhibition

## Abstract

Nucleoside analogues have excellent records as anti-HBV drugs. Chronic infections require long-term administration ultimately leading to drug resistance. Therefore, the search for nucleosides with novel scaffolds is of high importance. Here we report the synthesis of novel 2′-hydroxy- and 2′-hydroxymethyl-apionucleosides, **4** and **5**, corresponding triphosphates and phosphoramidate prodrugs. Triphosphate **38** of 2′-hydroxymethyl-apionucleoside 5 exhibited potent inhibition of HBV polymerase with an IC_50_ value of 120 nM. In an HBV cell-based assay, the phosphoramidate prodrug **39** demonstrated potent activity with an EC_50_ value of 7.8 nM.

## 1. Introduction

Hepatitis B is an infectious liver disease caused by the hepatitis B virus (HBV). An effective and safe vaccination has been available since 1982. However, in 2018, a total of 3322 cases of acute (short-term) hepatitis B were reported to CDC in the USA [1]. Since many people may not have symptoms or don’t know that they are infected, their illness is often not diagnosed so it remains unreported or uncounted. The CDC estimates that the actual number of acute hepatitis B cases was closer to 21,600 in 2018. Many more people (about 296 million) are estimated to be living with chronic, long-term hepatitis B worldwide [2]. HBV is an enveloped DNA virus that can cause both acute and chronic forms of the disease. Acute HBV infection is cleared out of the body usually in a few weeks, while chronic HBV infection can lead to permanent liver damage such as cirrhosis and liver cancer. Many nucleoside analogues are effective antiviral agents, with most of them targeting the reverse transcriptase activity of viral polymerases [3]. Six nucleoside derivatives (adefovir dipivoxyl, entecavir **1**, lamivudine, telbivudine, tenofovir alafenamide, and tenofovir disoproxil) and two interferons (alfa-2a/b and PEGylated alfa-2a/b) are currently approved for the treatment of hepatitis B in the United States [4]. Long-term administration of reverse transcriptase inhibitors is necessary, ultimately followed by the development of resistance [5]. The development of drug-resistant mutation to Lamivudine within five years is as high as 70%, while entecavir **1** has only a 1.2% of incidence [6]. Drug-resistant mutations to tenofovir were not reported initially, but mutations have been observed more recently [7]. Therefore, it remains important to search for new antiviral nucleoside analogues. 

**Figure 1:**
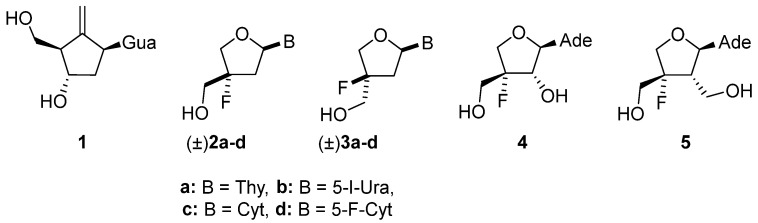
Structures of entecavir **1** and apionucleosides **2**–**5**.

A series of racemic 3′-fluoro-2′,3′-dideoxyapionucleosides (±)**2** and (±)**3** were found to inhibit HBV in vitro with EC_50_ values in the range of 0.011 μM for **2c** and 0.8 μM for both **2d** and **3b** (Figure 1) [8,9,10]. Asymmetric synthesis of stereoisomers was developed for **2a**,**c**, and **3a**,**c** [11,12,13]. In vitro anti HBV activity of separate enantiomers is reported only for **3a** (EC_50_ = 0.01 µM and 5.6 µM) [14]. We decided to synthesize and evaluate close analogues **4** and **5** bearing 2′-hydroxy and 2′-hydroxymethyl substituents respectively, in search of safe and potent nucleoside analogues for the treatment of HBV (Figure 1). 

## 2. Results and Discussion

**Scheme 1:**
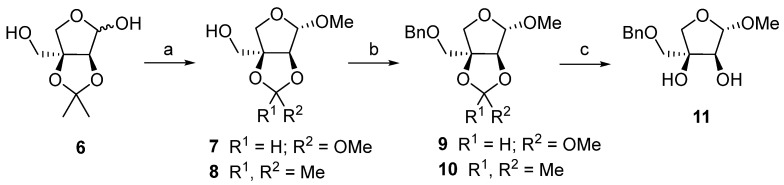
Reagents and conditions: (a) *p*TsOH⋅H_2_O, HC(OMe)_3_/MeOH, reflux, 6 h, 83% combined yield of **7** and **8**; (b) BnBr, NaH, TBAI, THF, rt, 24 h, 90% combined yield of **9** and **10**; (c) i. 80% AcOH, rt, 1 h; ii. NH_4_OH, rt, 30 min, 98%.

The synthesis of apionucleosides **4** and **5** started with compound **6**, which was prepared in four steps from D-ribose according to known procedures [15,16,17,18]. Formation of methyl glycoside under acidic conditions using *p*-toluenesulfonic acid provided methoxymethylene protected compound **7** and acetonide **8** in high overall yield (Scheme 1). The anomeric centers in both **7** and **8** formed with exclusive β-stereoselectivity. A mixture of compounds **7** and **8** was benzylated to provide **9** and **10**, respectively. Acidic treatment of the mixture resulted in diol **11**.

**Scheme 2:**
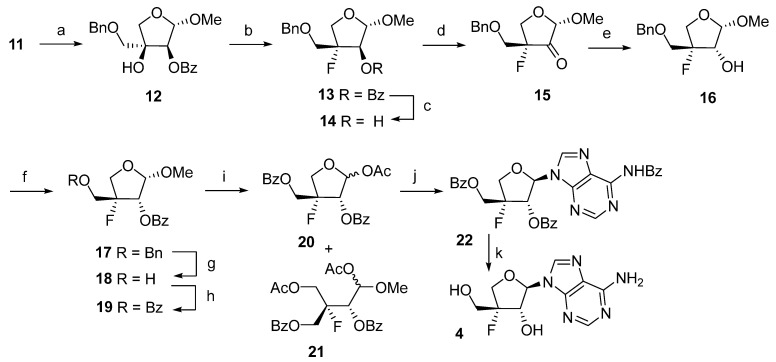
Reagents and conditions: (a) BzCl, pyridine, 0 °C, 2 h, 94%; (b) DAST, CH_2_Cl_2_, −65 to 0 °C, 2 h, 43%; (c) 7 M NH_3_ in MeOH, rt, overnight, 81%; (d) Dess-Martin periodinane, CH_2_Cl_2_, 0 °C to rt; 2 h; (e) NaBH_4_, MeOH, rt, overnight, 81% (two steps); (f) BzCl, pyridine, 0 °C to rt, 3 h, 88%; (g) Pd/C, H_2_, MeOH, rt, overnight, >99%; (h) BzCl, pyridine, 0 °C to rt, overnight, 93%; (i) H_2_SO_4_, AcOH, Ac_2_O, 0 °C, 3 h, 87% for **20**, 12% for **21**; (j) N^6^-benzoyladenine, BSA, TMSOTf, CH_3_CN, 65 °C, 3 h; (k) 7 M NH_3_ in MeOH, rt, overnight, 47% (two steps).

**Figure 2:**
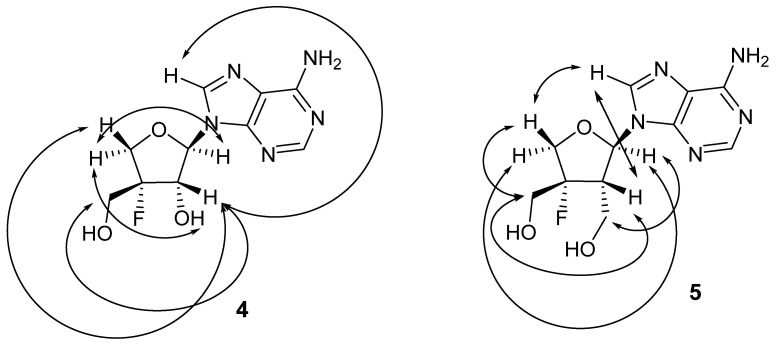
The observed proton NOE interactions (shown with arrows) important for the β-configuration determination of nucleosides **4** and **5**.

The apionucleoside **4** was synthesized as presented in Scheme 2. Secondary alcohol in **11** was benzoylated and the tertiary alcohol of **12** was transformed by nucleophilic fluorination with diethylaminosulfur trifluoride (DAST) to compound **13** in 43% yield [19]. The reversal of stereochemistry at C2 of **14** was achieved by a sequence of Dess-Martin oxidation to ketone **15** and following reduction with sodium borohydride to alcohol **16** [20]. Transformation of methoxy glycoside **17** to corresponding acetoxy glycoside under acidic conditions was plagued by concomitant deprotection of the benzyl group. Therefore, the benzyl group of **17** was exchanged by benzoyl in compound **19**. Acetolysis of **19** provided desired acetate **20** in high yield as an inconsequential mixture of two diastereomers. Small amounts of acyclic byproduct **21** were also isolated. Vorbrüggen condensation [21,22] of **20** with N^6^-benzoyladenine provided **22** which after debenzoylation afforded apionucleoside **4**. The stereochemistry of the glycosidic bond of **4** was confirmed by the 2D-NOESY experiment (Figure 2). The observed NOE contacts between H-8 of the base and H-2′ of the sugar in addition to NOE interactions between H-1′ and H-4′ confirmed β-orientation of the base. 

**Scheme 3:**
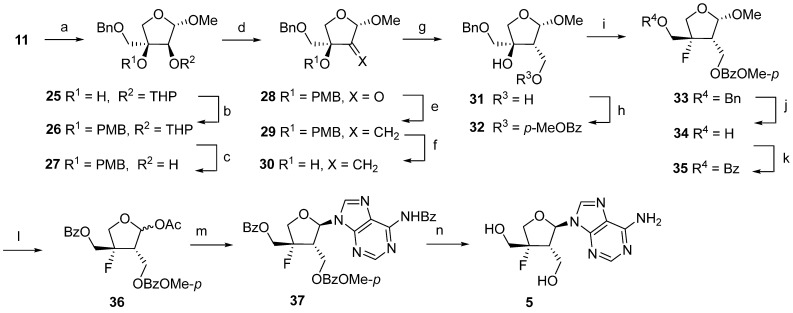
Reagents and conditions: (a) DHP, CSA, CH_2_Cl_2_, 0 °C, 1.5 h, 97%; (b) PMBCl, NaH, TBAI, DMF, rt, overnight, 79%; (c) 80% AcOH, THF, 45 °C, 2 h, 92%; (d) Dess-Martin periodinane, CH_2_Cl_2_, 0 °C to rt, 2.5 h, 98%; (e) PPh_3_CH_3_I, KHMDS, toluene, rt, 2 h, 69%; (f) DDQ, CH_2_Cl_2_/H_2_O, 0 °C, 1.5 h, 89%; (g) i. 9-BBN, THF, 40 °C, 24 h; ii. NaBO_3_·4H_2_O, EtOH/H_2_O, 50 °C, 3 h, 96%; (h) *p*-MeOBzCl, Et_3_N, DMAP, CH_2_Cl_2_, 0 °C to rt, 1 h, 93%; (i) DAST, CH_2_Cl_2_, –70 °C, 1 h, 20%; (j) Pd/C, H_2_, MeOH, rt, overnight, 91%; (k) BzCl, pyridine, 0 °C to rt, overnight, 96%; (l) H_2_SO_4_, AcOH, Ac_2_O, 0 °C, 2 h; (m) N^6^-benzoyladenine, BSA, TMSOTf, CH_3_CN, 65 °C, 3 h, 66%; (n) 7 M NH_3_ in MeOH, rt, 72 h, 84%.

The synthesis of 3′-fluoro-2′-hydroxymethyl apionucleoside **5** started with unsuccessful attempts to oxidize the secondary alcohol moiety of **11**. Therefore, a different synthetic approach was designed (Scheme 3). Tetrahydropyranyl protection of the secondary alcohol of **11** gave compound **25** in high yield as a mixture of two diastereomers. Protection of the tertiary alcohol with a 4-methoxybenzyl group (**26**) followed by THP deprotection afforded alcohol **27**. The Dess-Martin oxidation of **27** gave ketone **28** in excellent yield. The Wittig olefination of **28** provided alkene **29** in a 69% yield [23]. Removal of the PMB protecting group provided tertiary vinyl alcohol **30** in high yield [24,25]. Subsequent hydroboration of **30** with 9-BBN proceeded in very high yield and with exclusive stereoselectivity providing a single diastereoisomer of **31** [26,27,28]. Primary alcohol in **31** was protected with the 4-methoxybenzoyl group to give **32**. Electron-rich 4-methoxybenzoyl group was chosen because, as compared to the unsubstituted benzoyl group, it stabilizes the oxocarbenium ion by a more effective neighboring group participation during the glycosylation step [29]. In the next step, the tertiary alcohol of **32** was converted to fluoride **33** by using DAST. The yield of nucleophilic fluorination was low due to the formation of various elimination byproducts. Following hydrogenolysis of benzyl ether provided primary alcohol **34**, which was protected as a benzoyl ester in a straightforward way to give compound **35**. Acetolysis of a glycosidic bond in **35** gave acetate **36** as a mixture of two diastereomers together with some amounts of open chain byproduct; this mixture was used without separation in Vorbrüggen condensation with N^6^-benzoyladenine. After chromatography and deprotection, nucleoside **5** was obtained in good yield [30,31,32]. Stereochemistry was confirmed by 2D-NOESY NMR spectroscopy (Figure 2) and elemental composition by HRMS. The observed NOE contacts between H-8 of the base and H-4′ of the sugar confirmed the β orientation of the base. 

**Figure 3:**
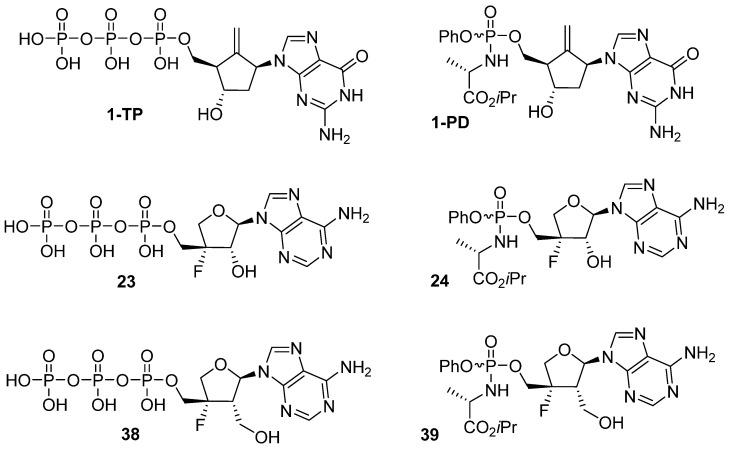
Structures of phosphoramidate prodrugs and tripohosphates of entecavir and synthesized apionucleosides.

A prerequisite for a nucleoside antiviral drug is the cellular activation of its triphosphate. Direct administration of the triphosphate is not possible due to the highly charged nature of phosphate moiety. Cellular bioactivation of nucleosides to triphosphates is often limited by the first phosphorylation step. To bypass this rate-limiting step, monophosphate prodrug technology has been applied [33,34,35]. We synthesized phosphoramidate prodrugs **24** and **39** as well as triphosphate **23** and **38** (Figure 3). 

Phosphoramidate of entecavir (**1-PD**) [36] and its triphosphate (**1-TP**) [37] were used as positive controls.

3′-Fluoroapionucleosides **4** and **5**, and their phosphoramidate prodrugs **24** and **39** were tested in cell-based assays against HBV activity. The data are summarized in Table 1. Neither **4** nor its prodrug **24** showed any anti-HBV activity. While 2′-hydroxymethyl substituted nucleoside **5** itself did not show any anti-HBV activity, its prodrug **39** exhibited strong inhibition of HBV in this cell-based assay with EC_50_ of 7.8 nM. This may be explained by the kinetically difficult first phosphorylation step of **5** in cells [35,36,37]. Prodrug **39** can be considered a masked monophosphate, thus overcoming the rate-limiting first phosphorylation step [21,33,34,35]. Moreover, prodrug **39** showed anti-HIV activity in a cell-based assay. The corresponding entecavir prodrug (**1-PD**) inhibited HBV replication with EC_50_ of 31 nM. None of the tested apionucleosides **4** and **5** or apionucleoside prodrugs **24** and **39** showed any cytotoxicity at their highest tested concentrations (Table 1). 3′-Fluoroapionucleoside triphosphates **23** and **38** were tested in HBV polymerase and HIV-1 reverse transcriptase (HIV-1 RT) enzymatic assays. The data in Table 1 shows that 2′-hydroxymethyl substituted triphosphate **38** had an IC_50_ of 0.12 µM with HBV polymerase, and it was a much more potent inhibitor of HIV-1 RT than 2′-hydroxy-substituted triphosphate **23** (IC_50_ of 0.32 µM vs. 4.2 µM). The triphosphate compound **38** was also tested with human DNA polymerases α, β, and γ, and it had an IC_50_ of 80 µM, >100 µM, and >100 µM, respectively, suggesting the 3′-fluoroapionucleoside triphosphate **38** has low or no inhibition activity against human DNA polymerases.

## 3. Materials and Methods

All commercially obtained solvents and reagents were used as received. All solvents used for chemical reactions were anhydrous grade unless specifically indicated. Structures of the target compounds in this work were assigned by use of NMR spectroscopy and MS spectrometry. The purities of all non-salt compounds were >95% as determined on an Agilent 1200 HPLC, XTerra 3.5 µm 4.6 × 150 mm MS C18 column, using 0.04% (*v*/*v*) TFA in water and 0.02% (*v*/*v*) TFA in acetonitrile as mobile phase. The purities of all nucleotides were >95%, determined on an Agilent 1100 HPLC, 50 mM TEAA in water, and 50 mM TEAA in acetonitrile as mobile phase. ^1^H-, ^19^F-, and ^13^C-NMR spectra were recorded on a Bruker Avance III (400 MHz) or a Varian 400MR (400 MHz) NMR spectrometer. Chemical shifts are reported in parts per million (ppm, *δ*) using the residual solvent line as an internal reference. Splitting patterns are reported as s (singlet), d (doublet), t (triplet), q (quartet), m (multiplet), or br s (broad singlet). Coupling constants (*J*) are reported in hertz (Hz). Mass spectrometric analyses for nucleosides were performed on an Agilent 1200 HPLC with Agilent 6110/6140/1956C MSD mass spectrometer using ESI as ionization, Phenomenex Luna C18 5 µm 5.0 × 20 mm column; mobile phase: 0.1% (*v*/*v*) TFA in water and 0.1% (*v*/*v*) TFA in acetonitrile, 40 °C, flow rate 0.4 mL/min. Mass spectrometric analyses for nucleotides were performed on an Agilent 1100 HPLC with API 2000 LC-MS/MS System using ESI as ionization, Synergi 75 × 2.0 mm, 4 µm Hydro-RP80Å column, 50 mM TEAA in water, and 50 mM in acetonitrile, flow rate 0.4 mL/min. Work-up procedures for most of the chemical reactions are the same or similar, therefore, unless specifically indicated the work-up refers to the following procedure: the reaction mixture at 0 °C is quenched with water, diluted with EtOAc or dichloromethane, washed with 5% sodium bicarbonate and then with brine, dried over anhydrous sodium sulfate, filtered, and concentrated to dryness. Purification on silica gel refers to flash chromatography on a silica gel column. HRMS spectra were measured on an Agilent G6230B Time-of-Flight mass spectrometer with Dual AJS (Agilent Jet Stream) ESI in positive mode coupled to an Agilent 1260 HPLC system, ACE 3 C18 35 × 2.1 mm column; mobile phase: acetonitrile and 0.1% (*v*/*v*) formic acid in water, 50 °C, flow rate 0.3 mL/min.

**(2*S*,3*S*,4*S*)-4-((Benzyloxy)methyl)-2-methoxytetrahydrofuran-3,4-diol (11).***p*-TsOH·H_2_O (456 mg, 2.4 mmol) was added to a solution of **6** [15,16,17,18] (15.4 g, 80.7 mmol) in methanol (665 mL) and trimethyl orthoformate (90 mL, 0.8 mol). After refluxing for 6 h the reaction mixture was stirred at rt overnight. Solid NaHCO_3_ (700 mg, 8.3 mmol) was added in portions until the mixture had neutral pH. The mixture was filtered, the filtrate was evaporated, and the residue was purified by column chromatography, 0–20% EtOAc in hexanes, to give colorless oil of **7** and **8** as **a** 2.5:1 mixture (13.8 g, 83%). TBAI (284 mg, 0.77 mmol) was added to a solution of **7** and **8** (15.8 g, 77 mmol) in anhydrous THF (160 mL). NaH (4.6 g, 115 mmol, 60% dispersion in mineral oil) was added in small portions and stirred at rt for 30 min, followed by BnBr (9 mL, 92 mmol). The reaction mixture was stirred at rt for 24 h. Florisil (6 g) was added, and the solvent was evaporated. The residue was dispersed in hexanes and insoluble material was filtered off and washed with additional quantities of hexanes. The combined filtrate was purified by column chromatography (hexane/EtOAc 1:3) to give 20.6 g (90% combined) of a mixture of **9** and **10** (46 and 22 mmol, respectively) as a colorless oil. The obtained mixture was dissolved in 80% aqueous acetic acid (50 mL), stirred for 1 h, and then neutralized with ammonium hydroxide (28–30%). After 30 min the solvents were evaporated. The residue was suspended in CH_2_Cl_2_, filtered, and the filtrate evaporated. The crude residue was purified by column chromatography with 25–80% EtOAc in hexanes to give 5.7 g of recovered **10** and 11.5 g of **11** (98%) as a colorless oil. ^1^H-NMR (CDCl_3_) δ = 3.01 (d, *J* = 5.2 Hz, 1H), 3.17 (s, 1H), 3.35 (s, 3H), 3.55 (d, *J* = 9.2 Hz, 1H), 3.61 (d, *J* = 9.6 Hz, 1H), 3.83 (d, *J* = 10.0 Hz, 1H), 3.88 (m, 1H), 3.90 (d, *J* = 10.4 Hz, 1H), 4.55 (d, *J* = 12.1 Hz, 1H), 4.59 (d, *J* = 11.7 Hz, 1H), 4.87 (d, *J* = 1.6 Hz, 1H), 7.26–7.36 (m, 5H). ^13^C-NMR (CDCl_3_) δ = 55.3 (q), 72.9 (t), 73.5 (t), 73.9 (t), 77.7 (d), 78.6 (s), 109.8 (d), 127.7 (d), 127.8 (d), 128.4 (d), 137.3 (s).

**(2*S*,3*S*,4*S*)-4-((Benzyloxy)methyl)-4-hydroxy-2-methoxytetrahydrofuran-3-yl benzoate (12).** Benzoyl chloride (2.35 mL, 20 mmol) was added to a solution of **11** (3.023 g, 11.9 mmol) in pyridine (40 mL) at 0 °C and for 2 h stirred at rt. Usual work-up and purification on silica gel with 10–60% EtOAc in hexanes afforded 4.02 g (94%) of **12** as a colorless oil. ^1^H-NMR (CDCl_3_) δ = 3.38 (s, 3H), 3.63 (d, *J* = 9.4 Hz, 1H), 3.77 (d, *J* = 9.4 Hz, 1H), 3.99 (d, *J* = 10.2 Hz, 1H), 4.01 (d, *J* = 9.8 Hz, 1H), 4.57 (d, *J* = 12.1 Hz, 1H), 4.63 (d, *J* = 12.1 Hz, 1H), 5.12 (d, *J* = 1.6 Hz, 1H), 5.28 (d, *J* = 1.6 Hz, 1H), 7.25–7.32 (m, 5H), 7.45 (t, *J* = 7.8 Hz, 2H), 7.56 (t, *J* = 7.4 Hz, 1H), 8.08 (d, *J* = 7.8 Hz, 2H). ^13^C-NMR (CDCl_3_): δ = 55.3, 73.2, 73.6, 74.4, 79.20, 79.24, 107.8, 127.6, 127.7, 128.37, 128.41, 129.4, 129.8, 133.3, 137.6, 165.7.

**(2*S*,3*R*,4*R*)-4-((Benzyloxy)methyl)-4-fluoro-2-methoxytetrahydrofuran-3-yl benzoate (13).** A solution of **12** (4.023 g, 11.2 mmol) in CH_2_Cl_2_ (25 mL) was added to DAST (4.4 mL, 33 mmol) in CH_2_Cl_2_ (60 mL) at −78 °C. The mixture was stirred at −65 °C for 1 h and at 0 °C for additional 1 h. The reaction mixture was quenched with saturated NaHCO_3_ solution. Usual work-up and purification on silica gel with 0–30% EtOAc in hexanes afforded 1.74 g (43%) of **13** as a colorless oil. ^1^H-NMR (CDCl_3_) δ = 3.45 (s, 3H), 3.80 (dd, *J* = 24.3, 11.4 Hz, 1H), 3.84 (dd, *J* = 21.9, 11.4 Hz, 1H), 4.23 (s, 1H), 4.29 (s, 1H), 4.55 (m, 2H), 5.00 (s, 1H), 5.54 (d, *J* = 15.6 Hz, 1H), 7.20–7.30 (m, 5H), 7.44 (m, 2H), 7.60 (m, 1H), 7.59 (m, 2H). ^19^F-NMR (CDCl_3_) δ = −159.44 (m).

**(2*S*,3*R*,4*R*)-4-((Benzyloxy)methyl)-4-fluoro-2-methoxytetrahydrofuran-3-ol (14).** Compound **13** (1.74 g, 4.8 mmol) was stirred in NH_3_ solution (50 mL, 7 M in MeOH) overnight at rt. The solvent was evaporated, and the crude reaction mixture was purified by column chromatography with 10–75% EtOAc in hexanes to give 1.0 g (81%) of **14** as a colorless oil. ^1^H-NMR (CDCl_3_) δ = 2.92 (d, *J* = 3.6 Hz, 1H), 3.41 (s, 3H), 3.81 (s, 1H), 3.87 (m, 1H), 3.98 (dd, *J* = 21.6, 10.4 Hz, 1H), 4.08 (dd, *J* = 23.2, 10.4 Hz, 1H), 4.36 (dd, *J* = 16.4, 3.6 Hz, 1H), 4.59 (d, *J* = 12.0 Hz, 1H), 4.68 (d, *J* = 12.0 Hz, 1H), 4.89 (s, 1H), 7.30–7.38 (m, 5H). ^19^F-NMR (CDCl_3_) δ = −158.87 (m).

**(2*S*,3*S*,4*R*)-4-((Benzyloxy)methyl)-4-fluoro-2-methoxytetrahydrofuran-3-yl benzoate (17).** Dess-Martin periodinane (2.050 g, 4.8 mmol) was added to a solution of **14** (728 mg, 2.8 mmol) in CH_2_Cl_2_ (20 mL) at 0 °C. After stirring for 1 h, the reaction mixture was warmed to rt and stirred for additional 1 h. Saturated NaHCO_3_ solution containing Na_2_S_2_O_3_ was added, and the reaction mixture was stirred vigorously for 2 h. The organic layer was separated and washed with brine, dried, and evaporated to give 765 mg of **15**. NaBH_4_ (215 mg, 5.7 mmol) was added to a solution of **15** in MeOH (20 mL) at 0 °C and the resulting mixture stirred for 6 h at rt, then 110 mg more (2.9 mmol) of NaBH_4_ was added and the mixture was left overnight at rt. The mixture was acidified with acetic acid to pH = 6 and evaporated. Usual work-up and purification on silica gel with 10–60% EtOAc in hexanes afforded 594 mg (81% over two steps) of **16** as a colorless oil. ^1^H-NMR (CDCl_3_) δ = 2.67 (dd, *J* = 12.0, 2.4 Hz, 1H), 3.47 (s), 3.63 (dd, *J* = 17.2, 10.2 Hz, 1H), 3.68 (dd, *J* = 11.4, 10.2 Hz, 1H), 4.04 (ddd, *J* = 19.2, 11.7, 4.7 Hz, 1H), 4.06–4.20 (m, 2H), 4.60 (m, 2H), 4.88 (d, *J* = 4.4 Hz, 1H). ^19^F-NMR (CDCl_3_) δ = −170.82 (m). Compound **16** (594 mg, 2.3 mmol) was treated as described for preparation of **12** to afford **17** (740 mg, 88%) of as a colorless oil. ^1^H-NMR (CDCl_3_) δ = 3.43 (s, 3H), 3.66–3.77 (m, 2H), 4.19 (s, 1H), 4.26 (s, 1H), 4.60 (m, 2H), 5.09 (dd, *J* = 19.6, 4.7 Hz, 1H), 5.26 (d, *J* = 4.7 Hz, 1H), 7.23–7.34 (m, 5H), 7.45 (m, 2H), 7.59 (m, 1H), 8.11 (m, 2H).

**(2*S*,3*S*,4*R*)-4-Fluoro-4-(hydroxymethyl)-2-methoxytetrahydrofuran-3-yl benzoate (18).** Pd/C (60 mg, 10% Pd basis) was added to a solution of **17** (630 mg, 1.8 mmol) in MeOH (10 mL) and stirred under a hydrogen atmosphere (1 atm) overnight at rt. The catalyst was filtered out, and the product was purified by column chromatography with 10–75% EtOAc in hexanes to give 496 mg (99%) of **18** as a colorless oil. ^1^H-NMR (CDCl_3_) δ = 2.34 (t, *J* = 6.6 Hz, 1H), 3.47 (s, 3H), 3.81 (ddd, *J* = 18.0, 12.1, 7.0 Hz, 1H), 3.91 (dt, *J* = 12.5, 6.3 Hz, 1H), 4.20 (dd, *J* = 12.2, 11.0 Hz, 1H), 4.26 (dd, *J* = 12.1, 11.0 Hz, 1H), 5.10 (dd, *J* = 18.9, 4.7 Hz, 1H), 5.24 (d, *J* = 4.7 Hz, 1H), 7.46 (m, 2H), 7.60 (m, 1H), 8.12 (m, 2H).

**((3*S*,4*S*,5*S*)-4-(Benzoyloxy)-3-fluoro-5-methoxytetrahydrofuran-3-yl)methyl benzoate (19).** Compound **18** (470 mg, 1.8 mmol) was treated as described for preparation of **12** to afford **19** (614 mg, 93%) as a colorless oil. MS, *m*/*z* 397 [M+Na^+^]. ^1^H-NMR (CDCl_3_) δ = 3.46 (s, 3H), 4.24 (dd, *J* = 23.5, 11.4 Hz, 1H), 4.34 (dd, *J* = 24.3, 11.4 Hz, 1H), 4.63 (m, 2H), 5.15 (dd, *J* = 18.4, 4.7 Hz, 1H), 5.33 (d, *J* = 4.7 Hz, 1H), 7.32 (m, 2H), 7.44 (m, 2H), 7.52 (m, 1H), 7.59 (m, 1H), 7.96 (m, 2H), 8.12 (m, 2H).

**(3*S*,4*S*)-2-Acetoxy-4-((benzoyloxy)methyl)-4-fluorotetrahydrofuran-3-yl benzoate (20)** and **(2*S*,3*S*)-4-acetoxy-2-(acetoxymethyl)-2-fluoro-4-methoxybutane-1,3-diyl dibenzoate (21).** Concentrated H_2_SO_4_ (44 µL) was added to a solution of 19 (614 mg, 1.6 mmol) in acetic acid (40 mL) and Ac_2_O (20 mL). After stirring for 3 h at 0 °C, the reaction mixture was poured into saturated NaHCO_3_ solution and stirred for 15 min. Usual work-up and purification on silica gel with 10–75% EtOAc in hexanes afforded 578 mg (87%) of 20 as an inseparable mixture of two diastereomers in a 1.5:1 ratio as colorless oil, followed by 93 mg (12%) of 21 as an inseparable mixture of two diastereomers in a 1:1 ratio as colorless oils. Complete stereochemistry of individual diastereomers of 20 and 21 was not assigned. 20: MS, *m*/*z* 425 [M+Na^+^]. ^1^H-NMR (CDCl_3_, signals of both diastereoisomers, detectable resonances of minor diastereomer marked with asterisk): δ = 2.10 (s, 6H), 4.29–4.84 (m, 8H), 5.48 (dd, *J* = 19.2, 4.8 Hz, 1H*), 5.60 (dd, *J* = 9.6, 2.0 Hz, 1H), 6.49 (dd, *J* = 2.0, 1.6 Hz, 1H), 6.60 (d, *J* = 4.8 Hz, 1H*), 7.33 (m, 2H*), 7.40–7.63 (m, 10H), 7.96 (m, 2H*), 8.04 (m, 2H), 8.05 (m, 2H*), 8.09 (m, 2H). ^19^F-NMR (CDCl_3_) δ = −169.97 (m), −175.11 (m). ^13^C-NMR (CDCl_3_, signals of both diastereoisomers, detectable resonances of minor diastereoisomer marked with asterisk): δ = 20.9 (q), 21.0 (q*), 64.3 (dt*, *J* = 30.5 Hz), 64.4 (dt, *J* = 27.5 Hz), 71.9 (dd*, *J* = 14.5 Hz), 73.6 (dt*, *J* = 25.9 Hz), 73.8 (dt, *J* = 25.2 Hz), 77.5 (dd, *J* = 14.5 Hz), 93.9 (d*), 95.6 (ds, *J* = 195.3 Hz), 98.1 (ds, *J* = 198.4 Hz), 128.37 (d*), 128.43 (d), 128.5 (d, 2C), 128.9 (s), 129.0 (s), 129.67 (d*), 129.74 (d), 129.9 (d*), 130.0 (d), 133.40 (d*), 133.44 (d), 133.72 (d*), 133.74 (d), 165.1 (s*), 165.3 (s), 165.6 (s*), 165.7 (s), 169.3 (s), 169.7 (s*). 21: MS, *m*/*z* 499 ([M+Na^+^]. ^1^H-NMR (CDCl_3_, signals of both diastereoisomers): δ = 1.89 (s, 3H), 2.08 (s, 3H), 2.09 (s, 3H), 2.11 (s, 3H), 3.49 (s, 3H), 3.50 (s, 3H), 4.48–4.81 (m, 8H), 5.77 (dd, *J* = 16.8, 6.3 Hz, 1H), 5.90 (dd, *J* = 11.0, 3.1 Hz, 1H), 6.13 (dd, *J* = 3.1, 1.2 Hz, 1H), 6.19 (d, *J* = 6.6 Hz, 1H), 7.46 (m, 8H), 7.56–7.63 (m, 4H), 8.01–8.09 (m, 8H). ^19^F-NMR (CDCl_3_) δ = −171.36 (m, 1F), −175.22 (m, 1F).

**(2*R*,3*S*,4*R*)-2-(6-Amino-9*H*-purin-9-yl)-4-fluoro-4-(hydroxymethyl)tetrahydrofuran-3-ol (4).***N*,*O*-Bis(trimethylsilyl)acetamide (850 µL, 3.5 mmol) was added to a solution of N^6^-benzoyladenine (416 mg, 1.7 mmol) in CH_3_CN (15 mL) and stirred for 20 min at 80 °C. A solution of **20** (637 mg, 1.6 mmol) in CH_3_CN (6 mL) was added followed with TMSOTf (350 µL, 1.9 mmol). The reaction mixture was stirred at 65 °C for 3 h, then cooled to rt. Usual work-up and purification on silica gel with 1–10% MeOH in CH_2_Cl_2_ afforded 1.0 g of a mixture of products, **22** being the major component. A solution of **22** (900 mg, 1.5 mmol) in NH_3_ (20 mL, 7 M in MeOH) was stirred at rt overnight. The solvent was evaporated, and the remaining residue was triturated with CH_3_CN to give 203 mg (47% over two steps) of pure **4** as a colorless solid. MS, *m*/*z* 270 [M+H^+^]. HRMS, *m*/*z* [C_10_H_13_FN_5_O_3_^+^]: calc. 270.0997; found 270.1009. ^1^H-NMR (DMSO-d_6_) δ = 3.64–3.81 (m, 2H), 4.08 (dd, *J* = 21.5, 11.0 Hz, 1H), 4.45 (dd, *J* = 37.2, 11.0 Hz, 1H), 5.00 (dt, *J* = 23.1, 7.6 Hz, 1H), 5.28 (t, *J* = 5.9 Hz, 1H), 5.86 (d, *J* = 7.0 Hz, 1H), 5.93 (dd, *J* = 7.6, 0.8 Hz, 1H), 7.30 (br s, 2H), 8.15 (s, 1H), 8.39 (s, 1H). ^19^F-NMR (DMSO-d_6_) δ = −178.21 (m). ^13^C-NMR (DMSO-d_6_) δ = 60.4 (dt, *J* = 27.6 Hz), 72.7 (dd, *J* = 16.9 Hz), 72.8 (dt, *J* = 23.0 Hz), 87.4 (d), 101.2 (d, *J* = 184.8 Hz), 119.3 (s), 140.2 (d), 149.6 (s), 152.8 (d), 156.1 (s).

**((3*S*,4*S*,5*R*)-5-(6-Amino-9*H*-purin-9-yl)-3-fluoro-4-hydroxytetrahydrofuran-3-yl)methyl triphosphate (23).** Dry nucleoside **4** (13 mg, 0.05 mmol) was dissolved in P(O)(OMe)_3_ (1 mL). *N*-methylimidazole (8 µL, 0.1 mmol) and POCl_3_ (10 µL, 0.1 mmol) were added at 0 °C and the reaction mixture was stirred for 30 min at rt. Tetrabutylammonium salt of pyrophosphate (150 mg) was added, followed by CH_3_CN (100 µL) to get a homogeneous solution. After stirring at rt for 1 h the reaction mixture was quenched with water and purified by ion-exchange chromatography (GE HiPrep Q HP, NaCl in 50 mM Tris solution, gradient from 0 to 1 M NaCl). The triphosphate **23** eluted at 0.75–0.8 M NaCl strength. The fractions containing **23** were concentrated and desalted by RP HPLC (Phenomenex Synergi 4 µm Hydro-RP 80 Å, water/CH_3_CN 1:0 gradient to 3:1, buffered with 50 mM triethylammonium acetate). The fractions containing **23** were concentrated and lyophilized three times to remove excess of buffer. MS, *m*/*z* 508 [M−H^−^]. ^1^H-NMR (D_2_O) δ = 4.24 (m, 3H), 4.53 (dd, *J* = 36.4, 11.7 Hz, 1H), 4.96 (dd, *J* = 21.5, 8.0 Hz, 1H), 6.00 (dd, *J* = 7.8, 1.2 Hz, 1H), 8.11 (s, 1H), 8.31 (s, 1H). ^19^F-NMR (D_2_O) δ = −176.83 (m). ^31^P-NMR (D_2_O) δ = −23.33 (t, *J* = 19.7 Hz), −11.77 (d, *J* = 19.7 Hz), −11.00 (d, *J* = 19.7 Hz).

**Isopropyl ((((3*S*,4*S*,5*R*)-5-(6-amino-9*H*-purin-9-yl)-3-fluoro-4-hydroxytetrahydrofuran-3-yl)methoxy)(phenoxy)phosphoryl)-*L*-alaninate (24).** Dry nucleoside **4** (20 mg, 0.074 mmol) was dissolved in a mixture of CH_3_CN (1.0 mL) and *N*-methylimidazole (0.50 mL). The reaction mixture was cooled to 0 °C and isopropyl (chloro(phenoxy)phosphoryl)-L-alaninate (68 mg, 0.22 mmol) in CH_3_CN (0.50 mL) was added. The reaction mixture was stirred at room temperature for 48 h, after which additional phosphorochloridate (113 mg, 0.37 mmol) was added and the reaction was stirred at 40 °C for 3 h. The reaction mixture was diluted with EtOAc and washed with water; organic fraction was dried with anhydrous Na_2_SO_4_. The solvent was evaporated, and the crude mixture was purified by column chromatography in 0–10% MeOH in DCM to give 24 contaminated with regioisomer. This mixture was further purified by reversed phase HPLC (Phenomenex Synergi 4 µm Hydro-RP 80 Å, water/CH_3_CN, gradient 30–80% MeCN, buffered with 50 mM triethylammonium acetate) to give 3 mg (7%) of pure 24 after lyophilization. Prodrug 24 was obtained as a mixture of Rp/Sp-isomers. MS, *m*/*z* 537 [M−H^+^]. ^1^H-NMR (CDCl_3_) δ = 1.22 (m, 12H), 1.38 (m, 6H), 3.99 (m, 4H), 4.23 (m, 4H), 4.47 (m, 6H), 4.98 (m, 4H), 5.94 (m, 2H), 5.98 (m, 4H), 7.10–7.33 (m, 10H), 7.87 (s, 1H), 7.91 (s, 1H), 8.22 (s, 1H), 8.23 (s, 1H). ^19^F-NMR (CDCl_3_) δ = −177.96 (m). ^31^P-NMR (CDCl_3_) δ = 2.70, 2.92.

**(3*S*,4*S*,5*S*)-3-((Benzyloxy)methyl)-5-methoxy-4-((tetrahydro-2*H*-pyran-2-yl)oxy)tetrahydrofuran-3-ol (25).** (±)-Camphorsulfonic acid (2.0 g, 8.7 mmol) was added to a solution of **11** (4.4 g, 17 mmol) and 3,4-dihydro-2*H*-pyran (7.9 mL, 87 mmol) in CH_2_Cl_2_ (110 mL) at 0 °C and stirred for 1.5 h. Solid anhydrous K_2_CO_3_ (4.8 g, 35 mmol) was added, and the reaction mixture was stirred for 1 h at rt. The mixture was diluted with CH_2_Cl_2_ (100 mL) and water (100 mL) and washed with saturated NaHCO_3_ solution (50 mL). Usual work-up and purification on silica gel with 10–60% EtOAc in hexanes afforded 5.7 g (97%) of **25** as an inseparable mixture of two diastereomers in a 1:1 ratio as a colorless oil. MS, *m*/*z* 361 [M+Na^+^]. ^1^H-NMR ( CDCl_3_) δ = 1.20–1.90 (m, 12H), 3.30–3.38 (m, 2H), 3.36 (s, 3H), 3.37 (s, 3H), 3.48–3.55 (m, 4H), 3.78–3.88 (m, 2H), 3.87–3.92 (m, 3H), 4.01 (dd, *J* = 9.8, 1.6 Hz, 1H), 4.06 (d, *J* = 2.0 Hz, 1H), 4.10 (d, *J* = 2.3 Hz, 1H), 4.53–4.63 (m+dd, *J* = 5.9, 2.3 Hz, 5H), 4.74 (t, *J* = 3.5 Hz, 1H), 4.93 (d, *J* = 2.3 Hz, 1H), 4.99 (d, *J* = 2.0 Hz, 1H), 7.25–7.37 (m, 10H). ^13^C-NMR (CDCl_3_) δ = 19.1, 20.1, 24.8, 25.1, 30.2, 30.6, 55.2, 62.5, 63.7, 72.3, 72.8, 73.4, 73.5, 74.1, 74.4, 78.5, 78.8, 82.8, 83.2, 99.5, 99.8, 108.8, 127.5, 127.62, 127.65, 128.2, 128.3, 137.8, 138.0.

**2-(((2*S*,3*S*,4*S*)-4-((Benzyloxy)methyl)-2-methoxy-4-((4-methoxybenzyl)oxy) tetrahydrofuran-3-yl)oxy)tetrahydro-2*H*-pyran (26).** A solution of **25** (6.25 g, 18 mmol) in anhydrous DMF (25 mL) was added to a suspension of NaH (1.1 g, 28 mmol, 60% dispersion in mineral oil) in DMF (75 mL) under an argon atmosphere and stirred at 40 °C for 1 h. The reaction mixture was cooled to 0 °C, TBAI (680 mg, 1.8 mmol) and 4-methoxybenzyl chloride (3.7 mL, 28 mmol) in DMF (15 mL) were added. The reaction mixture was stirred at rt overnight. Solvents were evaporated. Usual work-up and purification on silica gel with 10–60% EtOAc in hexanes gave 6.7 g (79%) of **26** as an inseparable mixture of two diastereomers in a 1:1 ratio as a colorless oil. Complete stereochemistry of individual diastereoisomers of **26** was not assigned. MS, *m*/*z* 481 [M+Na^+^]. ^1^H-NMR (CDCl_3_, signals of both diastereoisomers): δ = 1.40–1.85 (m, 12H), 3.37 (s, 3H), 3.39 (s, 3H), 3.41–3.53 (m, 2H), 3.62 (d, *J* = 9.8 Hz, 1H), 3.63 (d, *J* = 10.6 Hz, 1H), 3.66 (d, *J* = 10.2 Hz, 1H), 3.77 (d, *J* = 10.2 Hz, 1H), 3.79 (s, 6H), 3.83–3.94 (m, 2H), 4.00 (d, *J* = 10.2 Hz, 1H), 4.13 (m, 2H), 4.156 (d, *J* = 10.2 Hz, 1H), 4.158 (d, *J* = 2.0 Hz, 1H), 4.18 (d, *J* = 3.1 Hz, 1H), 4.52–4.66 (m, 7H), 4.75 (d, *J* = 11.0 Hz, 1H), 4.78 (m, 2H), 5.02 (d, *J* = 3.1 Hz, 1H), 5.08 (d, *J* = 2.0 Hz, 1H), 6.84 (m, 4H), 7.25–7.35 (m, 14H). ^13^C-NMR (CDCl_3_, signals of both diastereoisomers): δ = 19.3, 19.4, 25.28, 25.31, 30.4, 30.7, 55.2, 55.3, 55.5, 62.4, 62.5, 66.9, 70.8, 71.8, 72.5, 72.8, 73.4, 82.0, 82.2, 83.6, 84.4, 98.3, 98.8, 109.2, 109.7, 113.52, 113.54, 127.56, 127.62, 128.28, 128.30, 128.6, 128.9, 131.5, 131.7, 138.0, 138.1, 158.8.

**(2*S*,3*S*,4*S*)-4-((Benzyloxy)methyl)-2-methoxy-4-((4-methoxybenzyl)oxy)tetrahydrofuran-3-ol (27).** Acetic acid (100 mL, 80% in water) was added to a solution of **26** (6.74 g, 15 mmol) in THF (36 mL) and the reaction mixture was stirred at 45 °C for 2 h. The solvents were evaporated, and the crude product was purified by column chromatography with 10–60% EtOAc in hexane to give 5.1 g (92%) of **27** as a colorless oil. MS, *m*/*z* 397 ([M+Na^+^]. ^1^H-NMR (CDCl_3_): δ = 3.08 (d, *J* = 6.3 Hz, 1H), 3.36 (s, 3H), 3.72 (d, *J* = 10.6 Hz, 1H), 3.77 (d, *J* = 10.6 Hz, 1H), 3.80 (s, 3H), 3.91 (dd, *J* = 6.3, 2.0 Hz, 1H), 3.94 (d, *J* = 10.6 Hz, 1H), 4.14 (d, *J* = 10.2 Hz, 1H), 4.54 (d, *J* = 10.2 Hz, 1H), 4.56 (m, 2H), 4.60 (d, *J* = 10.6 Hz, 1H), 4.86 (d, *J* = 1.8 Hz, 1H), 6.87 (m, 2H), 7.24 (m, 2H), 7.28–7.40 (m, 5H). ^13^C-NMR (CDCl_3_) δ = 55.3, 55.4, 67.3, 70.6, 71.4, 73.6, 78.3, 83.4, 110.2, 113.9, 127.77, 127.85, 128.4, 129.3, 130.0, 137.7, 159.3.

**(2*S*,4*S*)-4-((Benzyloxy)methyl)-2-methoxy-4-((4-methoxybenzyl)oxy)dihydrofuran-3(2*H*)-one (28).** Dess-Martin periodinane (13 g, 30 mmol) was added to a solution of **27** (6.7 g, 18 mmol) in CH_2_Cl_2_ (240 mL) at 0 °C. After stirring for 30 min, the reaction mixture was warmed to rt and stirred for an additional 2 h. The solvent was evaporated, and the crude reaction mixture was dissolved in diethyl ether (300 mL), insoluble material was filtered off. The filtrate was stirred vigorously with saturated NaHCO_3_ solution containing Na_2_S_2_O_3_ for 30 min. The organic layer was separated, washed with brine, and dried. The solvent was evaporated to give 6.5 g (98%) of **28** that was used directly in the next step without further purification. ^1^H-NMR (CDCl_3_) δ = 3.46 (s, 3H), 3.64 (d, *J* = 10.6 Hz, 1H), 3.78 (d, *J* = 10.6 Hz, 1H), 3.79 (s, 3H), 4.33 (d, *J* = 11.0 Hz, 1H), 4.45 (d, *J* = 10.6 Hz, 1H), 4.48 (d, *J* = 10.2 Hz, 1H), 4.52 (d, *J* = 12.1 Hz, 1H), 4.55 (d, *J* = 11.0 Hz, 1H), 4.56 (d, *J* = 12.1 Hz, 1H), 4.76 (s, 1H), 6.84 (m, 2H), 7.20 (m, 2H), 7.26–7.36 (m, 5H). ^13^C-NMR (CDCl_3_) δ = 55.3, 55.8, 66.8, 67.9, 72.5, 73.8, 78.5, 98.8, 113.8, 127.7, 127.8, 128.4, 129.3, 129.7, 137.5, 159.3, 206.7.

**(2*S*,4*S*)-4-((Benzyloxy)methyl)-2-methoxy-4-((4-methoxybenzyl)oxy)-3-methylenetetrahydrofuran (29).** KHMDS (87 mL, 44 mmol, 0.5 M in toluene) was added to a solution of PPh_3_CH_3_I (21 g, 52 mmol) in toluene (50 mL). The mixture was stirred at 80 °C for 30 min, during which yellow colored solution ensued. It was transferred to a solution of **28** (6.4 g, 17 mmol) in toluene (50 mL). The reaction mixture was stirred at rt for 2 h, quenched with water and diluted with diethyl ether. The aqueous layer was separated and washed with diethyl ether (3×), combined organic fractions were washed with brine and dried with anhydrous Na_2_SO_4_. The solvents were evaporated, and the crude product was purified by column chromatography with 2–50% of EtOAc in hexanes to give 4.5 g (69%) of **29** as a colorless oil. ^1^H-NMR (CDCl_3_) δ = 3.40 (s, 3H), 3.59 (d, *J* = 10.2 Hz, 1H), 3.76 (d, *J* = 10.2 Hz, 1H), 3.79 (s, 3H), 4.16 (d, *J* = 10.6 Hz, 1H), 4.22 (d, *J* = 10.6 Hz, 1H), 4.36 (d, *J* = 11.0 Hz, 1H), 4.43 (d, *J* = 11.0 Hz, 1H), 4.55 (d, *J* = 12.1 Hz, 1H), 4.63 (d, *J* = 12.5 Hz, 1H), 5.34 (dd, *J* = 1.6, 1.2 Hz, 1H), 5.43 (d, *J* = 1.6 Hz, 1H), 5.54 (d, *J* = 1.2 Hz, 1H), 6.85 (m, 2H), 7.23 (m, 2H), 7.26–7.36 (m, 5H). ^13^C-NMR (CDCl_3_) δ = 54.7, 55.1, 65.2, 73.06, 73.08, 73.5, 83.3, 104.9, 113.5, 114.1, 127.4, 127.5, 128.2, 128.8, 130.7, 138.0, 147.6, 158.8.

**(3*S*,5*S*)-3-((Benzyloxy)methyl)-5-methoxy-4-methylenetetrahydrofuran-3-ol (30).** DDQ (4 g, 18 mmol) was added to a solution of **29** (4.4 g, 12 mmol) in CH_2_Cl_2_ (100 mL) and water (5 mL) at 0 °C and the mixture vigorously stirred for 1.5 h. Usual work-up and purification by silica gel chromatography with 10–75% EtOAc in hexanes gave 2.6 g (89%) of **30** as a colorless oil. MS, *m*/*z* 273 [M+Na^+^]. ^1^H-NMR (CDCl_3_) δ = 2.86 (s, 1H), 3.40 (s, 3H), 3.59 (m, 2H), 3.90 (d, *J* = 9.4 Hz, 1H), 4.08 (d, *J* = 9.8 Hz, 1H), 4.57 (d, *J* = 11.7 Hz, 1H), 4.62 (d, *J* = 12.1 Hz, 1H), 5.34 (m, 1H), 5.42 (d, *J* = 1.2 Hz, 1H), 5.49 (d, *J* = 1.6 Hz, 1H), 7.28–7.38 (m, 5H).

**(3*S*,4*R*,5*S*)-3-((Benzyloxy)methyl)-4-(hydroxymethyl)-5-methoxytetrahydrofuran-3-ol (31).** 9-BBN (45 mL, 22 mmol, 0.5 M in THF) was added to a solution of **30** (1.86 g, 7.4 mmol) in THF (10 mL) at rt under argon. After stirring for 24 h at 40 °C in a sealed vessel, NaBO_3_·4H_2_O (5.2 g, 34 mmol), EtOH (50 mL), and water (50 mL) were added, and the reaction mixture was stirred at 50 °C for 1.5 h. Another portion of NaBO_3_·4H_2_O (5 g, 32 mmol) was added and the reaction mixture was stirred at 50 °C for an additional 1.5 h, then acidified with acetic acid to pH = 6 and evaporated. The crude residue was dissolved in CH_2_Cl_2_, water was added, solids were filtered off, and the layers were separated. The aqueous layer was subsequently extracted with CH_2_Cl_2_ (2×). The combined organic extract was washed with brine and dried with anhydrous Na_2_SO_4_. Evaporated residue was purified on silica gel with 2–8% MeOH in CH_2_Cl_2_ to afford 1.9 g (96%) of **31** as a colorless oil. ^1^H-NMR (CDCl_3_) δ = 2.49 (dt, *J* = 6.6, 5.1 Hz, 1H), 2.73 (dd, *J* = 9.0, 4.3 Hz, 1H), 2.98 (s, 1H), 3.34 (s, 3H), 3.67 (d, *J* = 9.4 Hz, 1H), 3.73 (ddd, *J* = 12.1, 9.0, 5.5 Hz, 1H), 3.82 (d, *J* = 9.0 Hz, 1H), 3.84 (m, 1H), 3.85 (m, 2H), 4.58 (d, *J* = 11.7 Hz, 1H), 4.61 (d, *J* = 11.7 Hz, 1H), 5.13 (d, *J* = 5.5 Hz, 1H), 7.29–7.40 (m, 5H).

**((2*S*,3*R*,4*S*)-4-((Benzyloxy)methyl)-4-hydroxy-2-methoxytetrahydrofuran-3-yl)methyl 4-methoxybenzoate (32).** Triethylamine (4.6 mL, 33 mmol) was added to a solution of **31** (2.23 g, 8.3 mmol) in CH_2_Cl_2_ (20 mL). The mixture was cooled to 0 °C and 4-methoxybenzoyl chloride (2.2 mL, 17 mmol) was added followed by the addition of DMAP (1.014 g, 8.3 mmol). The reaction mixture was let warm to rt. The reaction was quenched with water and diluted with CH_2_Cl_2_. The usual work-up and purification on silica gel with 10–50% EtOAc in hexanes yielded 3.106 g (93%) of **32** as a colorless oil. MS *m*/*z* 425 ([M+Na^+^]. ^1^H-NMR (CDCl_3_) δ = 2.79 (ddd, *J* = 8.6, 6.6, 5.1 Hz, 1H), 3.07 (s, 1H), 3.30 (s, 3H), 3.64 (d, *J* = 8.6 Hz, 1H), 3.75 (d, *J* = 9.0 Hz, 1H), 3.86 (s, 3H), 3.89 (d, *J* = 9.8 Hz, 1H), 3.97 (d, *J* = 9.4 Hz, 1H), 4.40 (dd, *J* = 11.4, 8.6 Hz, 1H), 4.47 (dd, *J* = 11.0, 7.0 Hz, 1H), 4.54 (d, *J* = 11.7 Hz, 1H), 4.59 (d, *J* = 11.7 Hz, 1H), 5.09 (d, *J* = 4.7 Hz, 1H), 6.91 (m, 2H), 7.26–7.37 (m, 5H), 7.95 (m, 2H).

**((2*S*,3*S*,4*R*)-4-((Benzyloxy)methyl)-4-fluoro-2-methoxytetrahydrofuran-3-yl)methyl 4-methoxybenzoate (33).** Compound **33** (532 mg, 20%) as colorless oil was prepared from 2.5 g of **32** as described for the preparation of **13**. MS, *m*/*z* 427 [M+Na^+^]. ^1^H-NMR (CDCl_3_) δ = 2.65 (ddt, *J* = 26.6, 7.0, 5.1 Hz, 1H), 3.42 (s, 3H), 3.63 (dd, *J* = 18.8, 10.2 Hz, 1H), 3.74 (dd, *J* = 12.5, 10.2 Hz, 1H), 3.87 (s, 3H), 4.15 (m, 1H), 4.21 (m, 1H), 4.55 (m, 4H), 5.10 (d, *J* = 4.9 Hz, 1H), 6.90 (m, 2H), 7.26–7.37 (m, 5H), 7.94 (m, 2H). ^19^F-NMR (CDCl_3_): δ = −165.25 (ddq, *J* = 25.9, 19.1, 12.3 Hz).

**((2*S*,3*S*,4*R*)-4-Fluoro-4-(hydroxymethyl)-2-methoxytetrahydrofuran-3-yl)methyl 4-methoxybenzoate (34).** Compound **34** (376 mg, 91%) as colorless oil was prepared from 532 mg of **33** as described for the preparation of **18**. ^1^H-NMR (CDCl_3_) δ = 2.55 (ddt, *J* = 25.4, 7.0, 5.1 Hz, 1H), 3.43 (s, 3H), 3.75 (ddd, *J* = 21.9, 12.1, 7.8 Hz, 1H), 3.86 (s, 3H), 3.96 (ddd, *J* = 15.3, 12.1, 4.3 Hz, 1H), 4.16 (dd, *J* = 25.0, 11.0 Hz, 1H), 4.20 (dd, *J* = 23.9, 11.0 Hz, 1H), 4.53 (ddd, *J* = 11.7, 7.0, 1.6 Hz, 1H), 4.57 (dd, *J* = 11.4, 7.0 Hz, 1H), 5.11 (d, *J* = 5.1 Hz, 1H), 6.92 (m, 2H), 7.97 (m, 2H). ^19^F-NMR (CDCl_3_): δ = −168.98 (m).

**((2*S*,3*S*,4*S*)-4-((Benzoyloxy)methyl)-4-fluoro-2-methoxytetrahydrofuran-3-yl)methyl 4-methoxybenzoate (35).** Compound **35** (487 mg, 96%) as colorless oil was prepared from 376 mg of **34** as described for the preparation of **19**. ^1^H-NMR (CDCl_3_) δ = 2.70 (dddd, *J* = 25.0, 7.4, 6.3, 5.1 Hz, 1H), 3.44 (s, 3H), 3.85 (s, 3H), 4.24 (dd, *J* = 24.3, 10.6 Hz, 1H), 4.30 (dd, *J* = 24.6, 11.0 Hz, 1H), 4.52 (dd, *J* = 21.1 Hz, 11.7 Hz, 1H), 4.57–4.73 (m, 3H), 5.17 (d, *J* = 5.1 Hz, 1H), 6.91 (m, 2H), 7.44 (m, 2H), 7.58 (m, 1H), 7.97 (m, 2H), 8.03 (m, 2H). ^19^F-NMR (CDCl_3_) δ = −165.57 (m).

**((2*R*,3*S*,4*R*)-2-(6-Amino-9*H*-purin-9-yl)-4-fluorotetrahydrofuran-3,4-diyl)dimethanol (5).** Compound **35** (487 mg) was treated with H_2_SO_4_ (26 µL) in acetic acid (30 mL) and Ac_2_O (20 mL) as described for preparation of compound **20** to afford 527 mg of crude **36**. The crude acetyl glycoside **36** was condensed with protected adenine to yield nucleoside **37** (402 mg, 66% yield) as described for the synthesis of **22**. MS, *m*/*z* 626 [M+H^+^]. ^1^H-NMR (CDCl_3_) δ = 3.76 (s, 3H), 4.23 (dq, *J* = 26.6, 7.0 Hz, 1H), 4.43 (dd, *J* = 21.5, 11.0 Hz, 1H), 4.69 (ddd, *J* = 11.5, 8.2, 1.2 Hz, 1H), 4.75–4.93 (m, 4H), 6.33 (d, *J* = 7.4 Hz, 1H), 6.79 (m, 2H), 7.50 (m, 4H), 7.58–7.64 (m, 4H), 8.00 (m, 2H), 8.08 (s, 1H), 8.12 (m, 2H), 8.63 (s, 1H), 9.04 (br s, 1H). ^19^F-NMR (CDCl_3_) δ = −170.72 (m,). Protected nucleoside **37** was subject to ammonolysis as described for **4** to obtain 48 mg (84%) of **5** as a colorless solid. MS, *m*/*z* 282 ([M−H^+^]. HRMS *m*/*z* [C_11_H_15_FN_5_O_3_^+^]: calc. 284.1153; found 284.1166. ^1^H-NMR (DMSO-d6) δ = 3.30 (dq, *J* = 27.0, 7.4 Hz, 1H), 3.53 (m, 1H), 3.80 (m, 2H), 3.94 (dd, *J* = 16.0, 12.5 Hz, 1H), 4.09 (dd, *J* = 20.4, 10.6 Hz, 1H), 4.46 (dd, *J* = 36.8, 10.6 Hz, 1H), 4.77 (br s, 1H), 5.35 (br s, 1H), 6.10 (d, *J* = 7.4 Hz, 1H), 7.28 (br s, 2H), 8.15 (s, 1H), 8.32 (s, 1H). ^19^F-NMR (DMSO-d6): δ = −172.99 (m). ^13^C-NMR (DMSO-d6): δ = 50.6 (dd, *J* = 19.1 Hz), 57.2 (dt, *J* = 11.4 Hz), 62.2 (dt, *J* = 25.2 Hz), 75.4 (dt, *J* = 24.4 Hz), 87.9 (d), 105.3 (d, *J* = 182.1 Hz), 119.4 (s), 140.1 (d), 149.1 (s), 152.6 (d), 156.1 (s).

**((3*S*,4*S*,5*R*)-5-(6-Amino-9*H*-purin-9-yl)-3-fluoro-4-(hydroxymethyl)tetrahydrofuran-3-yl)methyl triphosphate (38).** 4-Methoxytriphenylmethyl chloride (47 mg, 0.15 mmol) was added to a solution of nucleoside **5** (39 mg, 0.14 mmol) in pyridine (3 mL) and the reaction mixture was stirred overnight at rt, an additional amount (5 mg, 0.02 mmol) of MMTrCl was added, and the reaction mixture was kept at rt for 6 more hours. The reaction was quenched with methanol, solvents were evaporated, and the crude mixture was purified by column chromatography with 0–12% in CH_2_Cl_2_ to give 66 mg (85%) of protected intermediate which was treated as described for the synthesis of **23**. The fractions containing triphosphate were concentrated to a volume of ~1 mL and treated with 80% formic acid to remove the 4-methoxytriphenylmethyl protecting group. Triphosphate **38** was isolated as described for **23**. MS *m*/*z* 522 [M−H^+^]. ^1^H-NMR (D_2_O) δ = 3.27 (dq, *J* = 26.8, 7.2 Hz, 1H), 3.73 (dd, *J* = 10.8, 7.2 Hz, 1H), 3.96 (dd, *J* = 12.0, 6.0 Hz, 1H), 4.21 (dd, *J* = 22.0, 11.2 Hz, 1H), 4.23–4.39 (m, 2H), 4.46 (dd, *J* = 35.2, 11.6 Hz, 1H), 6.23 (d, *J* = 8.0 Hz, 1H), 8.12 (s,1H), 8.34 (s, 1H). ^31^P-NMR (D_2_O) δ = −22.64 (t, *J* = 20.3 Hz, 1P), −11.64 (d, *J* = 19.6 Hz, 1P), −6.47 (d, *J* = 20.9 Hz, 1P).

**Isopropyl ((((3*S*,4*S*,5*R*)-5-(6-amino-9*H*-purin-9-yl)-3-fluoro-4-(hydroxymethyl) tetrahydro-furan-3-yl)methoxy)(phenoxy)phosphoryl)-*L*-alaninate (39).** Nucleoside 5 (80 mg, 0.3 mmol) was dissolved in a mixture of CH_3_CN (2 mL) and *N*-methylimidazole (0.8 mL). Isopropyl (chloro(phenoxy)phosphoryl)-L-alaninate (172 mg, 0.6 mmol) in CH_3_CN (0.5 mL) was added and the reaction mixture was stirred at room temperature for 2.5 h, quenched with water and evaporated. The residue was dissolved in CH_2_Cl_2_ (15 mL) and washed with 5% CH_3_CO_2_H solution (15 mL). The solvent was evaporated, and the crude mixture was purified by reversed phase HPLC (Phenomenex Kinetex 5 µm C18 100 Å, water/CH_3_CN, gradient 25–95% MeCN, buffered with 0.1% HCO_2_H) to give 22 mg (14%) of 39 as a mixture of Rp/Sp-isomers. MS, *m*/*z* 553 ([M+H^+^]. HRMS, *m*/*z* [C_23_H_31_FN_6_O_7_P^+^]: calc. 553.1970; found 553.1966. ^1^H-NMR (CDCl_3_): δ = 1.21 (d, *J* = 6.3 Hz, 3H), 1.23 (d, *J* = 6.3 Hz, 1H), 1.24 (d, *J* = 6.3 Hz, 6H), 1.36 (d, *J* = 7.4 Hz, 1H), 1.38 (d, *J* = 7.0 Hz, 1H), 3.41 (m, 2H), 3.65 (m, 1H), 3.74 (m, 1H), 3.90–4.07 (m, 6H), 4.21 (dd, *J* = 11.0, 6.3 Hz, 1H), 4.26 (dd, *J* = 11.0, 6.3 Hz, 1H), 4.41–4.64 (m, 6H), 4.99 (sept, *J* = 6.3 Hz, 1H), 5.03 (sept, *J* = 6.3 Hz, 1H), 5.59 (br s, 4H), 6.17 (d, *J* = 6.3 Hz, 1H), 6.21 (d, *J* = 6.3 Hz, 1H), 7.13–7.35 (m, 10H), 7.89 (s, 1H), 7.92 (s, 1H), 8.30 (s, 1H), 8.31 (s, 1H). ^19^F-NMR (CDCl_3_) δ = −172.16 (m, 1F), −171.95 (m, 1F). ^31^P-NMR (CDCl_3_) δ = 2.36 (s), 2.73 (s). ^13^C-NMR (CDCl_3_) δ = 20.8, 20.88, 20.93, 21.0, 21.7, 21.77, 21.79, 21.84, 50.5, 50.6, 51.8, 51.9, 52.0, 52.1, 58.4, 58.5, 58.6, 58.8, 66.9, 67.0, 67.18, 67.22, 69.0, 75.5, 75.7, 76.0, 89.2, 89.4, 101.4, 101.6, 103.3, 103.6, 119.9, 120.0, 120.25, 120.30, 120.34, 120.4, 125.3, 129.87, 129.91, 139.7, 139.8, 149.08, 149.14, 150.6, 150.7, 152.7, 155.4, 155.5, 173.27, 173.34, 173.4.

### 3.1. HBV Replication Assay with HepG2.117 Cells

Tet-regulated HBV expression HepG2.117 cell line was used in this study [38]. HepG2.117 cells (passage less than 25 passages) were cultured in DMEM/F12 50/50 medium (cat#10-092-CM, Corning, Glendale, AZ, USA) with 10% FBS (Corning, cat#35-011-CV), 250 µg/mL G418 Sulfate (Corning, cat#30-234-CI), 2 µg/mL Tetracycline (cat#T3325, Teknova, Hollister, CA, USA) and 1× Penicillin/Streptomycin (Corning, cat#30-002-CI). For each assay, cells were plated in an assay medium containing DMEM/F12 50/50 medium, 2% Tet-system approved FBS (cat#631106, Clontech, Mountain View, CA, USA), and 1× penicillin/streptomycin.

Determination of 50% inhibitory concentration (EC_50_) of compounds in HepG2.117 cells was performed by the following procedure. On the first day, cells were washed with PBS two times after trypsinizing the cells. Then cells were washed once with the assay medium. Cells were seeded at 30,000–35,000 cells per 100 µL per well in Biocoat collagen-coated flat bottom 96 well plates. After incubation of the cells in a 37 °C, 5% CO_2_ incubator for 4 h, 10 µL test compounds serially diluted in assay media were added to the cell plate. The final DMSO concentration was 1%. The cells were incubated at 37 °C for 96 h.

The antiviral activity was measured using a Real-Time quantitative polymerase chain reaction (RT qPCR) assay directly measuring the HBV viral DNA copy numbers from the supernatant of HepG2.117 cells. The HBV Core primers and probes used in qPCR: the core forward primer was 5′-CTGTGCCTTGGGTGGCTTT-3′; the core reverse primer was 5′- AAGGAAAGAAGTC AGAAGGCAAAA-3′; the core probe was 5′/FAM/AGCTCCAAA/ZEN/TCCTTTATAAGGGTCGA TGTCCATG/3IBFQ/-3′. The RT qPCR was run for 20 min at 95 °C and 20 min at 60 °C for each cycle, 40 cycles in total. HBV viral DNA copy numbers were normalized to the level observed in the absence of an inhibitor, which was defined as 100%. EC_50_ was defined as the concentration of compound at which the HBV viral DNA copy numbers from the HepG2.117 cells were reduced by 50% relative to its level in the absence of the compound. 

In parallel, cell cytotoxicity (CC_50_) against HepG2 cells was measured using a luminescent cell viability assay to determine the number of viable cells in the culture based on quantitation of the adenosine triphosphate (ATP) present after a 4-day incubation period. On the first day, HepG2 cells were seeded at 15,000 cells per 100 µL per well with assay media containing DEME (Corning, cat#10-013-CV), 3% FBS (Coning, cat#35-011-CV), 1× penicillin/streptomycin and 1× Non-essential Amino Acid in Biocoat collagen-coated 96-well flat bottom plates. Cells were incubated in a 37 °C, 5% CO_2_ incubator for 4 h before compound dosing. The compound dilution and dosing procedure were identical to that for the determination of anti-HBV activity. After 96 h incubation, CellTiter-Glo^®^ reagent (Promega, Madison, WI, USA) was added to each well and incubated for 10 min at RT. Luminescence was measured on a Victor X3 multi-label plate reader. Cell viability is normalized to the level observed in the absence of an inhibitor, which was defined as 100%. 

### 3.2. HIV Single Cycle Infection Assay

Twenty-four hours prior to infection, CEM human T lymphoblast cells (ATCC, Manassas, VA, USA) were plated in assay media (MEM supplemented with 10% FBS, 1% penicillin/streptomycin (all Mediatech, Manassas, VA, USA) and 1% DMSO (Sigma-Aldrich, St Louis, MO, USA)) at a density of 5 × 10^5^ cells/mL (5 × 10^4^ cells/well) in white 96-well plates. Serially diluted compounds were added to cells and incubated overnight in a 37 °C, 5% CO_2_ incubator. The following day, cells were infected with VSV-G pseudotyped HIV NL4-3, in which parts of the env and nef genes were replaced with Renilla-luciferase. Infected cells were incubated for 72 h in a 37 °C, 5% CO_2_ incubator. The viral inoculum was titrated to achieve a Renilla-luciferase signal of approximately 100-fold over the background. Antiviral activity of compounds was measured by the addition of 100 µL of Renilla-Glo^®^ reagent (Promega, Madison, WI, USA) to infected cells. After a 10-min incubation at room temperature, luminescence was measured on a Victor X3 multi-label plate reader (Perkin Elmer, Waltham, MA, USA). Cytotoxicity of compounds to uninfected parallel cell cultures was determined by the addition of 100 µL CellTiter-Glo^®^ reagent (Promega, Madison, WI, USA), and incubation for 10 min at room temperature. Luminescence was measured on a Victor X3 multi-label plate reader.

### 3.3. HIV-1 Reverse Transcriptase and HBV Polymerase Activity Assays

HIV-1 reverse transcriptase (HIV-1 RT) was purchased from Abcam (cat#ab63979). Recombinant HBV polymerase (Hepatitis B virus genotype D subtype ayw, full length) was cloned using the baculovirus system, expressed in sf9 cells, and purified with similar strategy and methods described by Lanford et al. [39]. A DNA primer (5′-CCGAGTAGTGTTGG-3′) was synthesized by IDTDNA and a 358-nt RNA template was synthesized in-house using Megascript T7 transcription kit (ThermoFisher, cat#AM1334). dNTPs were purchased from Thermo Fisher and ^3^H-dTTP from Perkin Elmer. Filter plates were purchased from Millipore (cat#MABN0V050) and microscint-20 was purchased from Perkin Elmer (cat#6013621).

The RNA-dependent DNA polymerization (RdDp) activity of HIV-1 RT was measured by the incorporation of radioactively labeled nucleotides by HIV-1 RT into acid-insoluble DNA products from the DNA primer primed RNA template. To test compound inhibition, the reactions were performed at 30 °C for 40 min in a reaction mixture containing reaction buffer (50 mM Tris-Cl, pH 7.5, 100 mM KCl, 12.5 mM MgCl_2_, 4 mM DTT), 1 nM HIV-1 RT, 0.1 µM DNA primer, 0.02 µM RNA template, 10% DMSO, 0.1 µM dATP, 1 µM dGTP, 0.1 µM dCTP, 0.32 µM ^3^H-dTTP, and compounds at various concentrations. The 50 µL reactions were performed in 96-well plates. The reactions were quenched with a 60 µL cold mixture of 20% (*w*/*v*) TCA and 0.5 mM ATP and incubated at 4 °C for 1 h. The reactions were loaded onto 96-well filter plates. The filters on plates were washed three times with 10% TCA and once with 70% ethanol on a Millipore plate wash station with vacuum applied. The filters on the plate were air-dried and 40 μL Microscint-20 was added to each well. The acid-precipitated tritiated DNA products retained on the filters were detected by a Trilux MicroBeta scintillation reader (Perkin Elmer).

The RdDp activity of HBV polymerase was measured similarly as described for the HIV-1 RT. To test compound inhibition effect, the reactions were performed at 30 °C for 120 min in a reaction mixture containing reaction buffer (50 mM Tris-Cl, pH 7.5, 100 mM KCl, 12.5 mM MgCl_2_, 4 mM DTT), 15 µg/mL polymerase, 0.5 µM DNA primer, 0.05 µM RNA template, 10% DMSO, 0.046 µM dATP, 0.057 µM dGTP, 0.017 µM dCTP, 0.32 µM ^3^H-dTTP, and compounds at various concentrations. 

All data were analyzed with GraphPad Prism. The compound concentration at which the enzyme-catalyzed rate was reduced by 50% (IC_50_) was calculated by fitting the data to the equation Y = % Min + (% Max − % Min) / (1 + 10^((logIC_50_-X)*h)), where Y corresponds to the percent inhibition to the enzyme activity, % Min is the residual inhibition activity without compound, % Max is the maximum inhibition of enzyme activity at saturating compound concentration, and X corresponds to the log of compound concentrations, and h is the hillslope. 

### 3.4. Human DNA Polymerase Assays

The DNA-dependent DNA polymerization (DdDp) activity of human DNA polymerases α, β, and γ was measured as described previously [40]. Briefly, the assays were run with 5 U/mL recombinant DNA polymerase α (CHIMERx, cat#1075-1), 0.5 U/mL recombinant DNA polymerase β (CHIMERx, cat#1077-1), or 0.1 μg/mL recombinant DNA polymerase γ (Abcam, cat#196066), 62 μg/mL activated calf thymus DNA, 50 mM Tris-HCl (pH 8.0), 60 mM KCl, 5 mM MgCl_2_, 4 mM dithiothreitol (DTT), 0.1 mg/mL BSA, 2 μM dCTP, 2 μM dATP, 2 μM dGTP, 0.05 μCi/μL ^3^H-dTTP (Perkin Elmer) and compounds at various concentrations at 30 °C for 2 h. The 50 μL reactions were processed following the same filter plate method as described above for HIV-1 RT. Acid-precipitated tritiated DNA products retained on the filters were detected by a Trilux MicroBeta scintillation reader (Perkin Elmer). All data were analyzed as described above for HIV-1 RT.

## 4. Conclusions

We have synthesized two novel 3′-fluoro-2′-substituted apionucleoside analogues with adenine nucleobase. Phosphoramidate prodrug of 2′-hydroxymethyl analogue **39** showed promising inhibition against HBV with an EC_50_ of 7.8 nM. None of the synthesized nucleoside analogues and their respective prodrugs exhibited any cytotoxicity at their highest tested concentrations. 3′-Fluoroapionucleoside triphosphate **38** had an IC_50_ of 0.12 µM against HBV polymerase, and it was also a potent inhibitor of HIV-1 RT. Triphosphate **38** demonstrated low or no activity against human DNA polymerases.

## Figures and Tables

**Table 1:** Antiviral activities of 3′-fluoroapionucleosides, nucleotides triphosphates, and nucleoside phosphoramidate prodrugs.

Compound	HBV EC_50_ (μM)	HBV CC_50_ (μM)	HIV EC_50_ (μM)	HIV CC_50_ (μM)	HBV pol IC_50_ (μM)	HIV-1 RT IC_50_ (μM)
**4**	>100	>100	>33	>33	-	-
**23** (NTP)	-	-	-	-	n/a	4.2 (1)
**24**	>10	> 10	>33	>33	-	-
**5**	>10	>10	>33	>33	-	-
**38** (NTP)	-	-	-	-	0.12 (1)	0.32 (1)
**39**	0.0078 ± 0.0035 (3)	>10	0.3 (1)	>33	-	-
**1-TP**	-	-	-	-	0.014 ± 0.003 (11)	0.32 (1)
**1-PD**	0.031 ± 0.020 (3)	>10	>20	>20	-	-
**1**	0.0030 ± 0.0024 (38)	>100	1.24 ± 0.65 (2)	15.3 ± 1.2 (2)	-	-

Values of EC_50_, CC_50_, and IC_50_ are expressed as means ± SDs, followed by numbers of independent experiments in parentheses.

## Data Availability

Not applicable.

## Sample Availability

Samples of the compounds are not available from the authors.

## Abbreviations

Ade: adenine; Ac, acetyl; B, nucleobase; 9-BBN, 9-borabicyclo [3.3.1]nonane; Bn, benzyl; BSA, N,O-bis(trimethylsilyl)acetamide; Bz, benzoyl; Cyt, cytosine; CC50, 50% cytotoxic concentration, CSA, camphorsulfonic acid; DAST, N,N-diethylaminosuflur trifluoride; DDQ, 2,3-dichloro-5,6-dicyano-1,4-benzoquinone; DHP, 3,4-dihydro-2H-pyran; DMAP, 4-dimethylaminopyridine; DMF, N,N-dimethylformamide; DNA, deoxyribonucleic acid; EC50, half maximal effective concentration; Et, ethyl; ESI, electrospray ionization; HBV, hepatitis B virus; HIV, human immunodeficiency virus; HPLC, high-performance liquid chromatography; HRMS, high resolution mass spectrometry; IC50, half maximal inhibitory concentration; KHMDS, potassium bis(trimethylsilyl)amide; Me, methyl; MMTr, 4-methoxytriphenylmethyl; MS, mass spectrometry; NMR, nuclear magnetic resonance; PEG, polyethylene glycol; Ph, phenyl; PMB, 4-methoxybenzyl; pol, polymerase; RT, reverse transciptase; Thy, thymine; TBAI, tetra-n-butylammonium iodide; THF, tetrahydrofuran; THP, tetrahydropyran-2-yl; TMSOTf, trimethylsilyl trifluoromethanesulfonate; pTsOH, 4-methylbenzene-1-sulfonic acid; Ura, uracil.
